# Diaryl Disulfides as Novel Stabilizers of Tumor Suppressor Pdcd4

**DOI:** 10.1371/journal.pone.0151643

**Published:** 2016-03-16

**Authors:** Tobias Schmid, Johanna S. Blees, Magdalena M. Bajer, Janine Wild, Luca Pescatori, Giuliana Cuzzucoli Crucitti, Luigi Scipione, Roberta Costi, Curtis J. Henrich, Bernhard Brüne, Nancy H. Colburn, Roberto Di Santo

**Affiliations:** 1 Institute of Biochemistry I, Faculty of Medicine, Goethe-University Frankfurt, 60590, Frankfurt, Germany; 2 Dipartimento di Chimica e Tecnologie del Farmaco, Istituto Pasteur – Fondazione Cenci Bolognetti, “Sapienza” University of Rome, 00185, Rome, Italy; 3 Molecular Targets Laboratory, Center for Cancer Research, National Cancer Institute-Frederick, Frederick, MD, 21702, United States of America; 4 Basic Science Program, Leidos Biomedical Research, Inc., Frederick National Laboratory for Cancer Research, Frederick, MD, 21702, United States of America; 5 Laboratory of Cancer Prevention, Center for Cancer Research, National Cancer Institute-Frederick, Frederick, MD, 21702, United States of America; University of California at Davis, UNITED STATES

## Abstract

The translation inhibitor and tumor suppressor Pdcd4 was reported to be lost in various tumors and put forward as prognostic marker in tumorigenesis. Decreased Pdcd4 protein stability due to PI3K-mTOR-p70^S6K1^ dependent phosphorylation of Pdcd4 followed by β-TrCP1-mediated ubiquitination, and proteasomal destruction of the protein was characterized as a major mechanism contributing to the loss of Pdcd4 expression in tumors. In an attempt to identify stabilizers of Pdcd4, we used a luciferase-based high-throughput compatible cellular assay to monitor phosphorylation-dependent proteasomal degradation of Pdcd4 in response to mitogen stimulation. Following a screen of approximately 2000 compounds, we identified 1,2-bis(4-chlorophenyl)disulfide as a novel Pdcd4 stabilizer. To determine an initial structure-activity relationship, we used 3 additional compounds, synthesized according to previous reports, and 2 commercially available compounds for further testing, in which either the linker between the aryls was modified (compounds 2–4) or the chlorine residues were replaced by groups with different electronic properties (compounds 5 and 6). We observed that those compounds with alterations in the sulfide linker completely lost the Pdcd4 stabilizing potential. In contrast, modifications in the chlorine residues showed only minor effects on the Pdcd4 stabilizing activity. A reporter with a mutated phospho-degron verified the specificity of the compounds for stabilizing the Pdcd4 reporter. Interestingly, the active diaryl disulfides inhibited proliferation and viability at concentrations where they stabilized Pdcd4, suggesting that Pdcd4 stabilization might contribute to the anti-proliferative properties. Finally, computational modelling indicated that the flexibility of the disulfide linker might be necessary to exert the biological functions of the compounds, as the inactive compound appeared to be energetically more restricted.

## Introduction

The tumor suppressor Programmed cell death 4 (Pdcd4) is lost in a number of different tumors such as lung, colon, breast, ovarian, and pancreatic cancer [1,2]. Loss of Pdcd4 enhances neoplastic transformation, activator protein 1 (AP-1) transactivation, intravasation, and invasion *in vitro* [3,4] and Pdcd4-deficient mice appear more susceptible to the two stage skin carcinogenesis model, whereas transgenic overexpression of Pdcd4 decreased papilloma incidence and multiplicity in this model [5,6]. In addition, Pdcd4-deficient mice were shown to spontaneously develop lymphoma restricting their life span [7]. On the molecular level, Pdcd4 inhibits translation rather than transcription by interfering with the activity of the RNA helicase eukaryotic initiation factor (eIF) 4A through competition with the scaffold protein eIF4G [8]. Interestingly, in contrast to most tumor suppressors, Pdcd4 appears not to be inactivated mutationally [9]. Instead there is mounting evidence that Pdcd4 expression in tumors is predominantly controlled at a post-transcriptional level. In addition to the microRNA-21-dependent repression of Pdcd4 expression [10], increased proteasomal degradation contributes to the regulation of Pdcd4 levels in response to mitogens and inflammatory tumor environments [6,11,12]. Mechanistically, Pdcd4 protein contains a 70-kDa ribosomal protein S6 kinase 1 (p70^S6K1^) consensus phosphorylation sequence directly followed by the binding motif for the E3-ubiquitin ligase β-transducin repeat-containing protein (β-TrCP). Activation of p70^S6K1^ in response to mitogens such as the phorbol ester 12-*O*-tetradecanoylphorbol-13-acetate (TPA) results in phosphorylation of Pdcd4, followed by binding of β-TrCP, polyubiquitination, and subsequent proteasomal degradation of Pdcd4 [6,11].

Since stabilization of Pdcd4 emerged as an interesting concept for novel tumor therapeutics, we set out to identify novel Pdcd4 stabilizers. Previously, we already identified a number of Pdcd4 stabilizers [13–17]. Yet, these compounds were all natural products generally characterized by rather complex structures not easy to use as scaffolds to perform structure-activity relationship studies. Therefore, we decided to screen an in-house chemical synthetic library available in our laboratories. Here we present the identification of a novel group of Pdcd4 stabilizers providing the basis for an initial structure-activity relationship determination.

## Materials and Methods

### Reagents

All chemicals were purchased from Sigma-Aldrich (Schnelldorf, Germany) if not indicated otherwise. Rapamycin and TPA (12-*O*-tetradecanolyphorbol-13-acetate) were purchased from LC Laboratories (Woburn, MA, USA).

### Chemistry

Melting points were determined on a Bibby Stuart Scientific SMP1 melting point apparatus and are uncorrected. Infrared spectra were recorded on a Perkin-Elmer Spectrum-one spectrophotometer. ^1^H NMR and ^13^C NMR spectra were recorded at 400 MHz and 100 MHz, respectively, on a Bruker AC 400 Ultrashield 10 spectrophotometer (400 MHz). Dimethylsulfoxide-*d*_*6*_ 99.9% (code 44,139–2) and deuterochloroform 98.8% (code 41,675–4) of isotopic purity (Aldrich) were used. Column chromatographies were performed on a silica gel (Merck; 70–230 mesh) column. All compounds were routinely checked on thin layer chromatography using aluminum-baked silica gel plates (Fluka DC-Alufolien Kieselgel 60 F254). Developed plates were visualized by UV light. Solvents were reagent grade and, when necessary, were purified and dried by standard methods. Concentration of solutions after reactions and extractions involved the use of rotary evaporator (Büchi) operating at a reduced pressure (ca. 20 Torr). Organic solutions were dried over anhydrous sodium sulfate (Merck). All reactions were carried out under nitrogen; all solvents were freshly distilled under nitrogen and stored over molecular sieves for at least 3 h prior to use.

Compounds **1** (1,2-bis(4-chlorophenyl)disulfide) [18,19], **2** (4-chloro-*N*-(4-chlorophenyl)benzamide) [20], **3** ((*E*)-1,2-bis(4-chlorophenyl)diazene), and **4** (1,2-bis(4-chlorophenyl)hydrazine) [21] were synthesized as previously reported in the literature. Compounds **5** (1,2-bis(4-methoxyphenyl)disulfide) and **6** (1,2-bis(4-nitrophenyl)disulfide) were purchased from Sigma-Aldrich.

### Cell culture

HEK293 cells were purchased from ATCC-LGC Standard GmbH (Wesel, Germany). HEK293 Pdcd4-luc cells were maintained in DMEM supplemented with 10% FBS, 100 U/mL penicillin, 100 μg/mL streptomycin, 2 mM L-glutamine, and 3 μg/mL blasticidin as previously described [14,15]. Cells were cultivated in a humidified atmosphere with 5% CO_2_ at 37°C. Medium, supplements, and FBS came from PAA (Linz, Austria).

### Pdcd4 stabilization assay

Pdcd4 stabilization was assessed using a luciferase-based assay as previously described [15]. Briefly, HEK293 cells stably expressing either Pdcd4_(39–91)_luc or Pdcd4_(mut39-91)_luc were seeded in a 96-well plate (1 x 10^4^/well) and allowed to attach for 18 h before treatment. After appropriate incubations, cells were harvested in luciferase lysis buffer (25 mM Tris, 2 mM DTT, 1% Triton-X-100, 10% glycerol, pH 7.8) and frozen at -20°C for at least 2 h. After lysis at room temperature, luminescence was measured using *firefly* luciferase substrate solution (20 mM tricine, 2.67 mM 4MgCO_3_*Mg(OH)_2_*5H_2_O, 1.07 mM MgSO_4_*7H_2_O, 100 μM EDTA, 33.3 mM DTT, 530 μM ATP, 0.213 mg/mL coenzyme A, 470 mM D-luciferin) on a Mithras LB 940 (Berthold, Bad Wildbad, Germany). Pdcd4_(39–91)_luc expressing cells were used to determine Pdcd4 stabilization and the stabilizing activity of test samples was calculated using the following formula based on the relative light units (RLU) measured:

$$\text{Pdcd}4_{\left(39-91\right)}\;\text{stabilization}\;\left[\%\right]\;=\;\left(\text{RL}{\text{U}}_{\text{Cpd}}-\;\text{RL}{\text{U}}_{\text{TPA}}\right)\;/\;\left(\text{RL}{\text{U}}_{\text{DMSO}}-\;\text{RL}{\text{U}}_{\text{TPA}}\right)\;\times \;100.$$

Pdcd4_(mut39-91)_luc expressing cells served as a specificity control and effects were calculated according to the following formula:

$$\text{Pdcd}4_{\left(\text{mut}39-91\right)}\;\text{RLU}\;\left[\%\right]\;=\;\text{RL}{\text{U}}_{\text{Cpd}}\;/\;\text{RL}{\text{U}}_{\text{TPA}}\;\times \;100.$$

### Proliferation assay

HEK293 cells (1 x 10^4^/well), seeded in 96-well plates one day prior to the experiment, were treated as indicated and proliferation was analyzed over a time course of 5 days using an IncuCyte^®^ Live-Cell Imaging System (Essen Bioscience). Briefly, proliferation was assessed as increase in confluency of the cell layer by automated real-time, live-cell imaging every 4 h using the IncuCyte. Mean values of 3 wells per treatment were determined for at least 3 independent experiments.

### Cell cycle analysis

HEK293 cells (8 x 10^4^/well), seeded in 6-well plates one day prior to the experiment, were treated as indicated. Cell cycle distribution was assessed by 7-Amino-Actinomycin D (7-AAD) staining. Briefly, after treatment the cells were permeabilized using PBS ^+^ (PBS supplemented with 1.1 g/l sucrose, 0.5 mM EDTA and 50 μg/ml RNase A). Stainings were carried out in PBS^+^, 10% NP-40, and 5% 7-AAD (BD Pharmingen) and detected on a LSRFortessa flow cytometer (BD Biosciences).

### Cell death detection

HEK293 cells (8 x 10^4^/well), seeded in 6-well plates one day prior to the experiment, were treated as indicated. Viability was assessed using Annexin V/7-AAD co-stainings. Briefly, after treatment the cells were harvested in PBS and directly stained with 2% Annexin V-FITC (ImmunoTools) and 1% 7-AAD. Measurements were carried out on a LSRFortessa flow cytometer.

### Computational study

Conformational analyses were carried out using the software VEGAZZ rel.3.0.5.12. The systematic approach was chosen to explore the conformational space around the three rotatable bonds (σ1, σ2, σ3), with 60° step increments. For each compound all the 216 generated conformations were minimized using SP4 force field, and the energy was plotted against the number of the conformation.

### Statistical analysis

At least three independent experiments were performed. Data are presented as means ± SEM. Statistical analyses were performed using Student’s t-test.

## Results and Discussion

**Fig 1:**
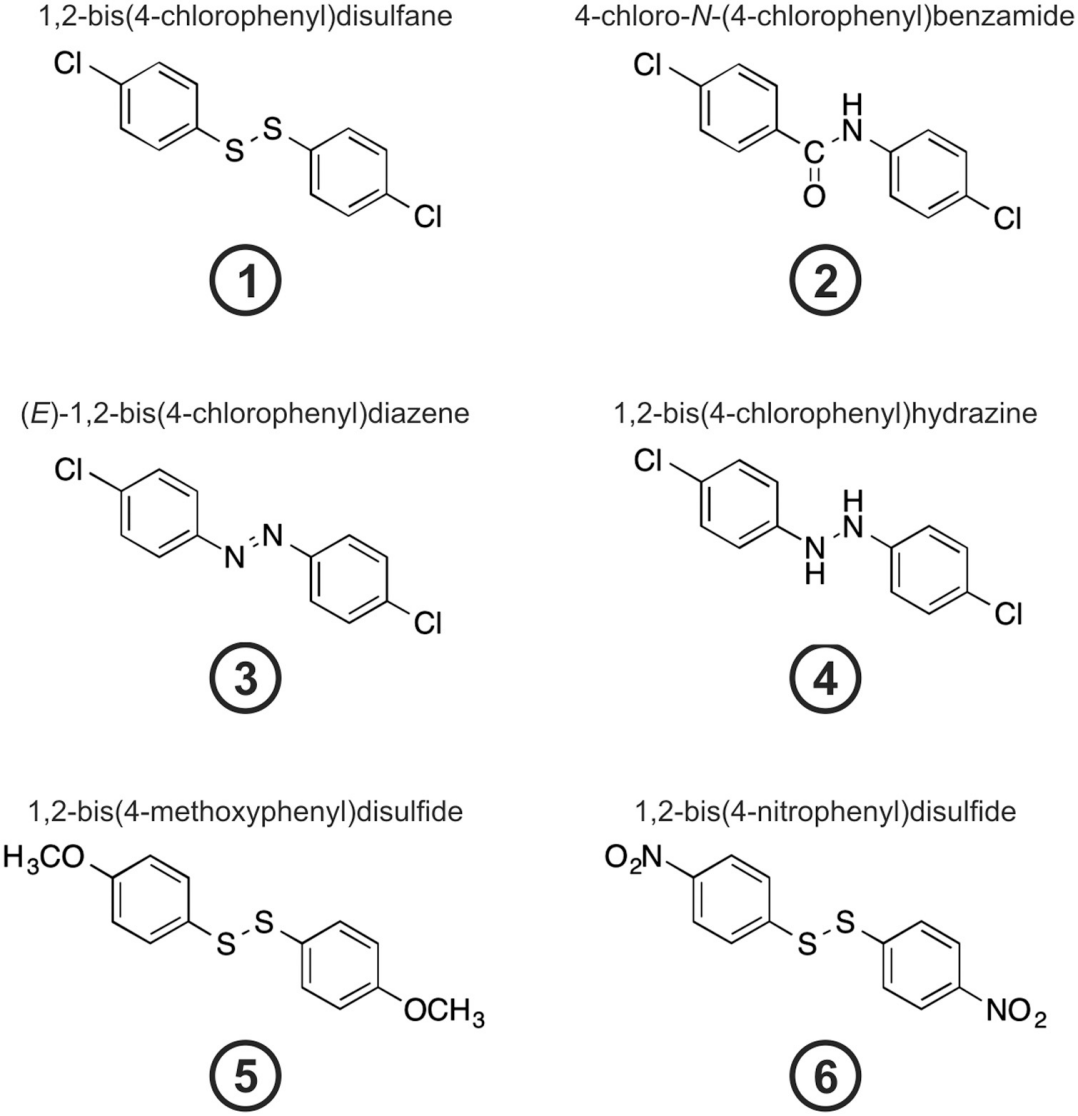
Chemical structures. Structures of compounds **1** (1,2-bis(4-chlorophenyl)disulfide), **2** (4-chloro-*N*-(4-chlorophenyl)benzamide), **3** ((*E*)-1,2-bis(4-chlorophenyl)diazene), **4** (1,2-bis(4-chlorophenyl)hydrazine), **5** (1,2-bis(4-methoxyphenyl)disulfide), and **6** (1,2-bis(4-nitrophenyl)disulfide).

We previously presented a high-throughput compatible, cell-based assay to identify compounds stabilizing Pdcd4 from TPA-induced degradation [13–15]. Hereto, we introduced a vector containing the phosphorylation-dependent degradation domain of Pdcd4 (aa 39–91) fused to luciferase (Pdcd4_(39-91)_luc) into HEK293 cells to follow TPA-induced degradation of Pdcd4. A vector where the critical serine residues 67, 71, and 76 within the phospho-degron were mutated to alanines (Pdcd4_(mut39–91)_luc) served as a TPA-insensitive control [14]. Importantly, due to the nature of the assay observed effects can be attributed solely to altered Pdcd4 protein stability, while mechanisms affecting Pdcd4 synthesis, e.g. via microRNA-21-dependent reduction of Pdcd4 mRNA stability or translation, can be excluded since these would not affect the reporter vector as the respective mRNA-target sequences are not present. Screening of a chemical library of approximately 2000 compounds including compounds with antifungal, antiviral, or antitumor activities, as well as their synthetic intermediates, we identified 1,2-bis(4-chlorophenyl)disulfide (**1**) (Fig 1) to stabilize Pdcd4 from TPA-induced degradation with a maximal Pdcd4 stabilizing activity at 10 μM. As structure and synthesis of this diaryl disulfide were previously described [18,19], we aimed at generating related compounds that could provide further insights into the structure-activity relationship concerning Pdcd4 stabilization.

First we decided to modify the linker between the aryl moieties of **1**. Thus, the amide **2**, the azo derivative **3**, and the corresponding hydrazine **4** were synthesized as previously reported in the literature [20,21]. We further included two commercially available derivatives, in which the chlorine atoms of **1** were replaced by either electron donating methoxy groups (**5**) and electron withdrawing NO_2_ group (**6**) for further analyses. Thus, a total of 6 compounds was analyzed in this study (Fig 1).

### Determination of the Pdcd4 stabilizing activities

**Fig 2:**
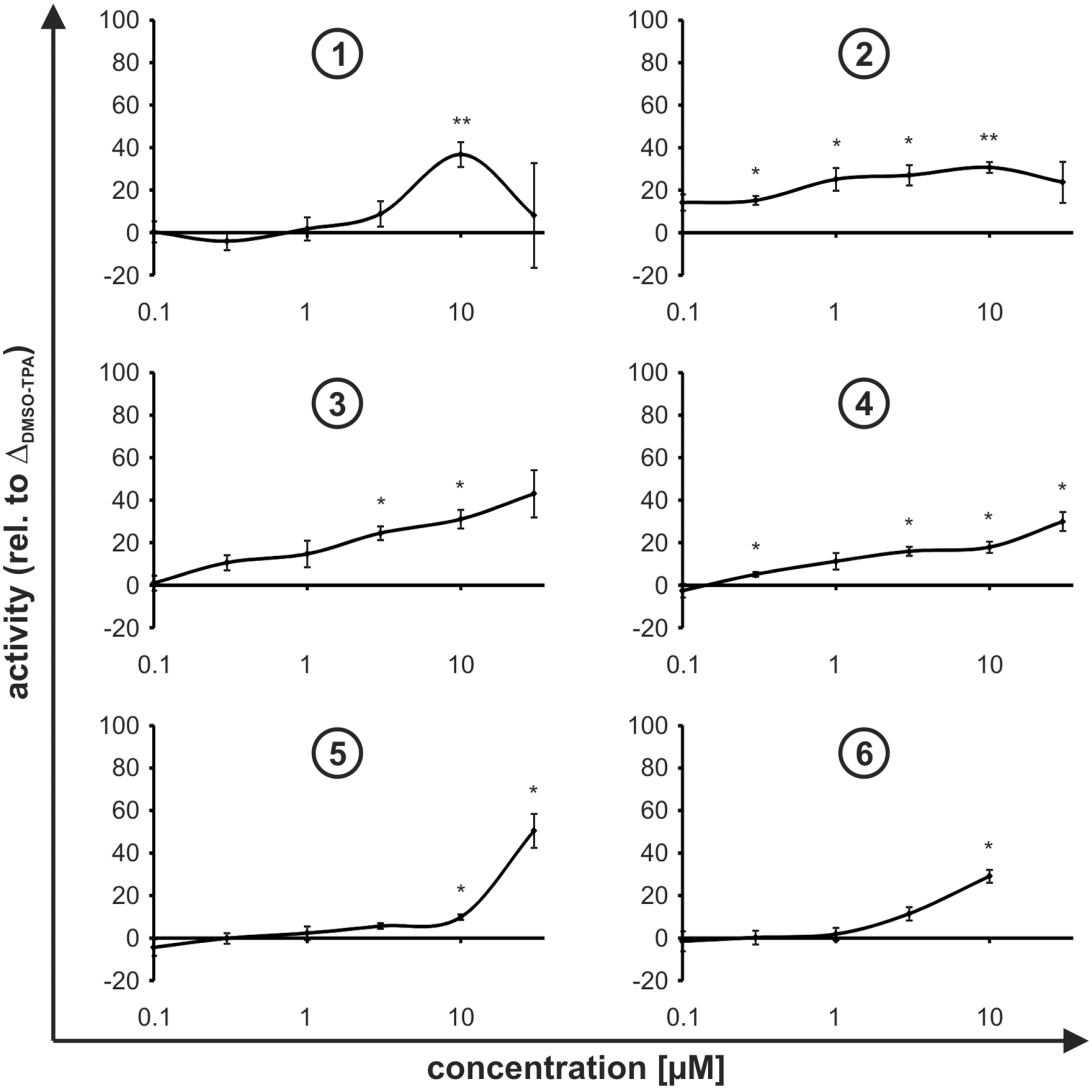
Pdcd4 stabilizing activity. HEK293 cells stably expressing the Pdcd4 stability reporter Pdcd4_(39–91)_luc were incubated for 8 h with TPA [10 nM] in combination with increasing concentrations (0.1–30 μM) of compounds **1**, **2**, **3**, **4**, **5**, and **6**. Pdcd4 stabilizing activity was determined relative to Δ(RLU_DMSO_–RLU_TPA_). Data are presented as means ± SEM of at least 3 independent experiments (* p<0.05, ** p<0.01, *** p<0.01).

We initially tested all compounds for their ability to stabilize Pdcd4. To this end, we co-treated Pdcd4_(39–91)_luc expressing stability reporter cells with TPA (10 nM) and compounds **1**, **2**, **3**, **4**, **5**, or **6** (0.1 to 30 μM) for 8 h. Compound **6** was only used up to 10 μM due to insolubility at higher concentrations. The Pdcd4 stabilizing activity was determined relative to the difference between DMSO-treated control and TPA-treated cells, where DMSO-treated cells was set as 100% and TPA-treated cells as 0%. Recovery of luciferase activity was considered as Pdcd4 stabilization as previously described [15]. In line with our previous observation, freshly synthesized compound **1** showed maximal Pdcd4 stabilization at 10 μM, with a sharp loss of activity at 30 μM (Fig 2). Compound **2** showed slightly elevated luciferase levels across all concentrations tested, whereas the luciferase signal steadily increased in response to compounds **3** and **4**. Compound **5** showed some Pdcd4 stabilization at the highest concentrations used (30 μM), whereas compound **6** showed enhanced activity starting at 3 μM (Fig 2).

**Fig 3:**
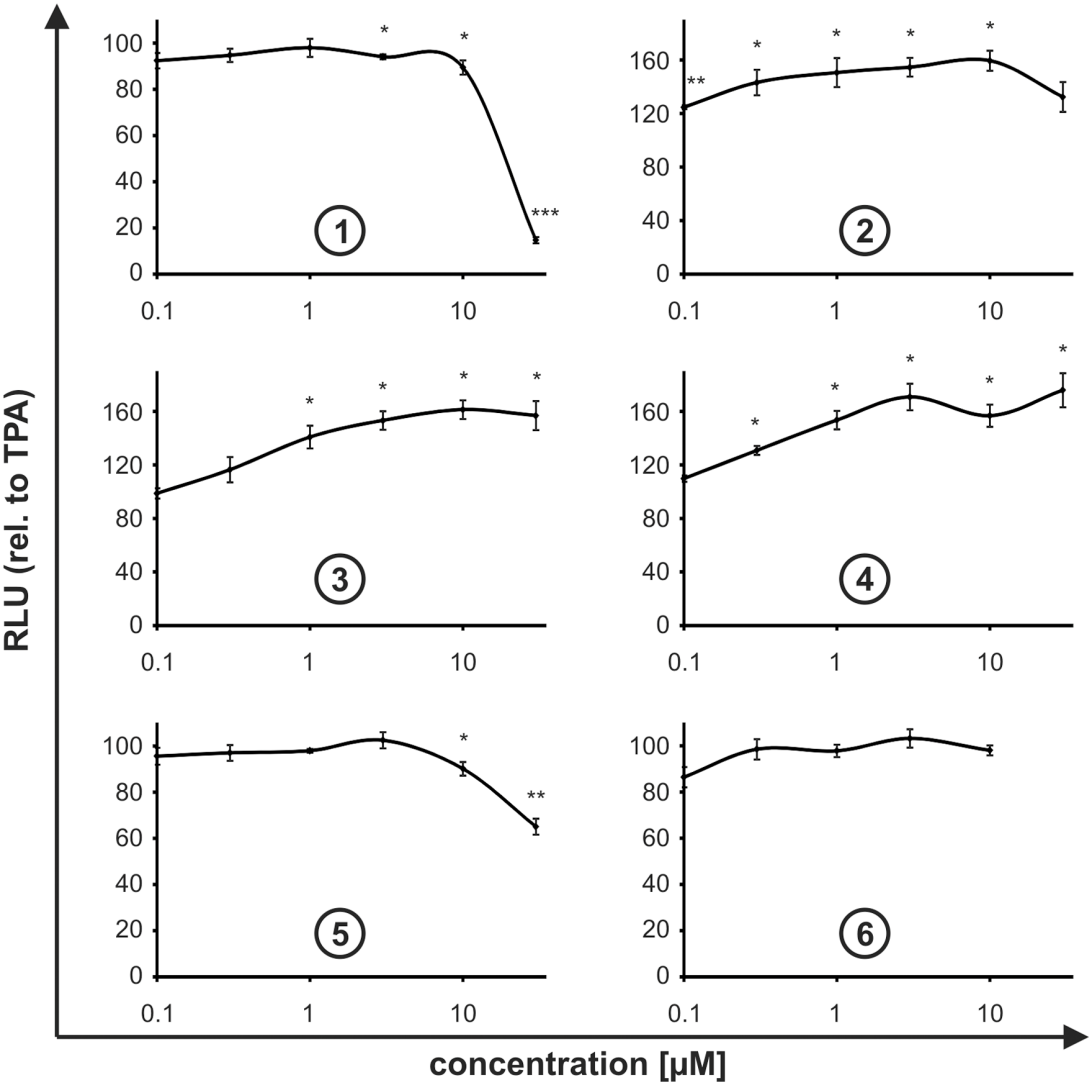
Nonspecific activity. HEK293 cells stably expressing Pdcd4_(mut39–91)_luc were incubated for 8 h with TPA [10 nM] in combination with increasing concentrations (0.1–30 μM) of compounds **1**, **2**, **3**, **4**, **5**, and **6**. Luciferase activity is given relative to TPA-treated controls. Data are presented as means ± SEM of at least 3 independent experiments (* p<0.05, ** p<0.01, *** p<0.01).

To evaluate the specificity of the observed activities, we treated the TPA-insensitive Pdcd4_(mut39–91)_luc expressing cell line in the same manner as the Pdcd4 stability reporter cell line. Since TPA did not alter the luciferase activity of this reporter as compared to DMSO, all compounds were evaluated relative to TPA-only as they were administered in combination with TPA (10 nM). Compound **1** did not affect the luciferase activity up to 10 μM. Yet, at 30 μM of compound **1** luciferase activity was markedly reduced (Fig 3). The loss of luciferase activity at 30 μM corresponded well with the marked reduction of the activity at 30 μM observed in the stability reporter cell line (Fig 2), suggestive for toxicity and/or nonspecific luciferase inhibition to overcome the Pdcd4 stabilizing capacity.

Compounds **2**, **3**, and **4** displayed either stable, but elevated luciferase levels (**2**) or a steady increase (**3**, **4**) in luciferase activity in the specificity control cells (Fig 3), following the same pattern as observed in the Pdcd4 stability reporter cells (Fig 2). As the luciferase activities behaved similarly in control and Pdcd4 stability reporter cells, these effects are likely to be attributable to a general effect on luciferase activity rather than to Pdcd4 stabilization by compounds **2**, **3**, and **4**.

In contrast, compounds **5** or **6** did not show elevated luciferase activities in the mutant cell line. Thus, the suggested Pdcd4 stabilizing activities observed for these compounds appeared not to be due to nonspecific effects. In the case of compound **5**, higher concentrations (10 and 30 μM) even slightly inhibited the luciferase signal (Fig 3), where the Pdcd4 stabilization was still observed. Thus, while the elevated Pdcd4 stabilizing activity (Fig 2) was not overcome by the general luciferase inhibition, this might cause an underestimation of the real stabilization potential of compound **5**.

In summary, in addition to compound **1**, compounds **5** and **6** appeared to stabilize Pdcd4 without affecting the specificity luciferase reporter. In the case of compounds **1** and **5**, the loss of luciferase activity at 30 μM might be an indicator for toxicity. Increasing toxicity associated with elevated Pdcd4 levels does not come as a surprise, since Pdcd4 was previously shown to contribute to cell death [22]. Compounds **2**, **3**, and **4** affected both reporter constructs similarly, i.e. lacked specific Pdcd4 stabilizing activity.

Taken together these data suggest that compound **1**, and to a lesser extend compounds **5** and **6**, contain concentration-dependent Pdcd4 stabilizing activity. Thus, while replacement of the chlorine atoms with different substituents (compounds **5** and **6**) only slightly modulated the Pdcd4 stabilizing activity of the parent compound **1**, altering the linker between the two phenyl rings of **1** by substituting the disulfide by an amide function, a diazo group, or a hydrazine moiety (compounds **2**, **3**, and **4**, respectively) completely abolished the activity.

### Determination of the proliferation, cell cycle, and viability inhibitory capacities

**Fig 4:**
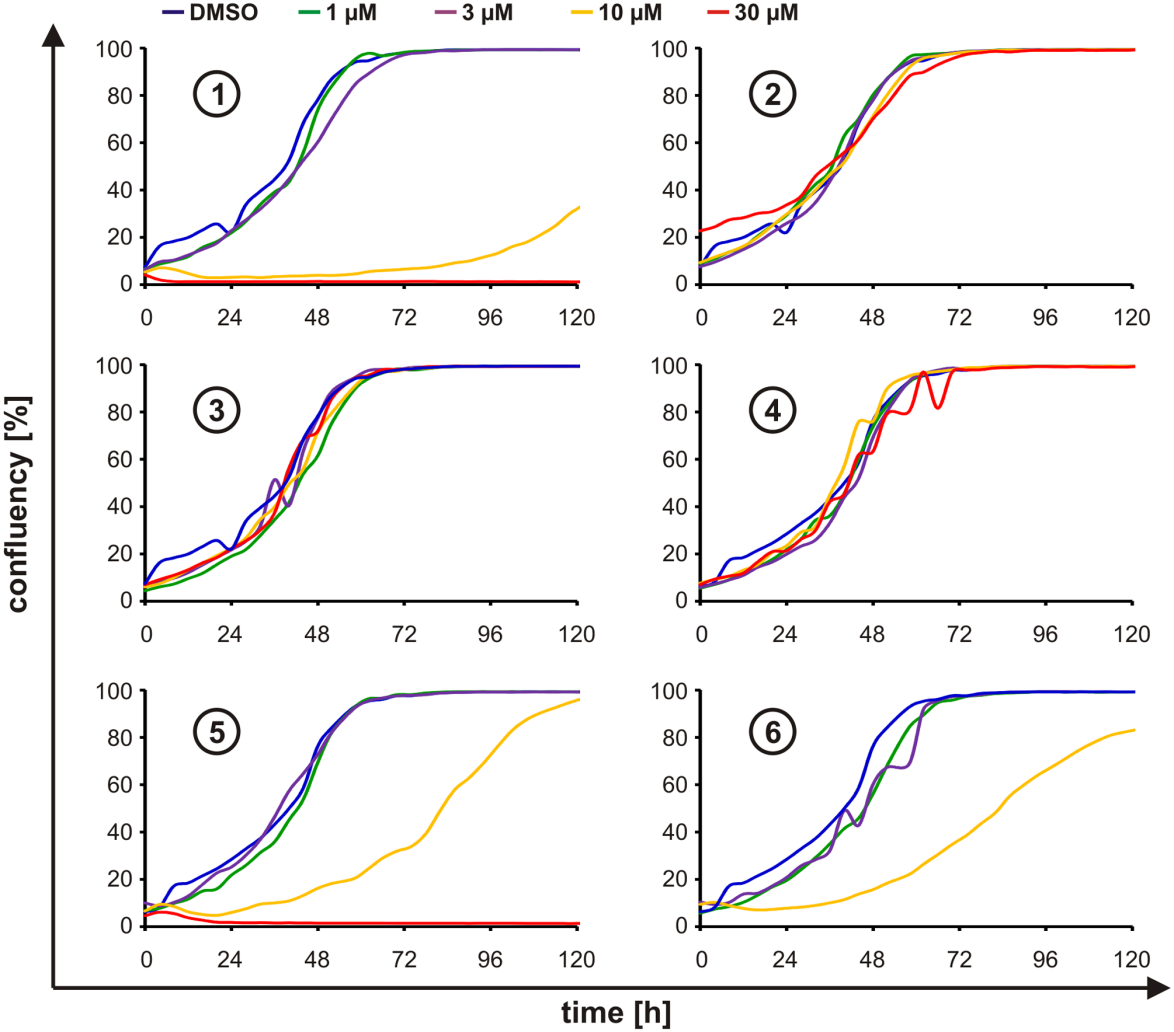
Proliferation. HEK293 cells stably expressing Pdcd4_(39–91)_luc were incubated for 5 days with DMSO (blue) or increasing concentrations of compounds **1**, **2**, **3**, **4**, **5**, and **6** (1 μM (green), 3 μM (violet), 10 μM (yellow), and 30 μM (red)). Confluency was assessed every 4 h in an IncuCyte instrument. Proliferation kinetics shown are representative for at least 3 independent experiments and represent means of 3 wells treated in parallel (* p<0.05, ** p<0.01, *** p<0.01).

While the anti-tumorigenic properties of Pdcd4 were primarily attributed to its transformation inhibitory function [23], Pdcd4, as the name implies, was tightly linked with the induction of apoptosis as well [22,24]. Since we observed that the most potent Pdcd4 stabilizing compounds **1** and **5** elicited a marked loss of luciferase activity at 30 μM in target as well as off-target cells, which could be indicative for a loss of viability, we aimed at determining the impact of these compounds on cell proliferation. To this end, HEK293 cells were plated at low densities and their confluency was followed every 4 hours for 5 days *via* automated microscopy in an IncuCyte instrument in the presence or absence of compounds (1–30 μM). In line with the lacking Pdcd4 stabilization, compounds **2**, **3**, and **4** did not affect proliferation, i.e. proliferation was not altered at any concentration as compared to the control (Fig 4). In contrast, the three compounds exerting Pdcd4 stabilizing activity (compounds **1**, **5**, and **6**) markedly inhibited proliferation starting at concentrations of 10 μM (Fig 4, yellow lines). While this correlated well with the concentrations effectively stabilizing Pdcd4 for compounds **1** and **6** (Fig 4), compound **5** showed only a minimal Pdcd4 stabilizing activity at this concentration. In line with their differences in Pdcd4 stabilizing potency, compound **1** appeared to be more effective in blocking proliferation at 10 μM. At 30 μM (red lines), where compound **6** could not be tested, compounds **1** and **5** completely inhibited proliferation, yet still allowing for the detection of Pdcd4 stabilizing properties at least in the case of compound **5**. This observation further corroborates the above-described finding where the luciferase activity of the off-target cell line was inhibited by 85% at a concentration of 30 μM of compound **1**, whereas compound **5** inhibited nonspecific luciferase only by 35% at 30 μM (Fig 3). While the correlation between Pdcd4 stabilization and anti-proliferative effects appeared to be rather weak in the case of compound **5**, the different exposure times need to be kept in mind, i.e. while in the luciferase-based assays compound-exposure was 8 h, the proliferation assay was carried out for 5 d.

**Fig 5:**
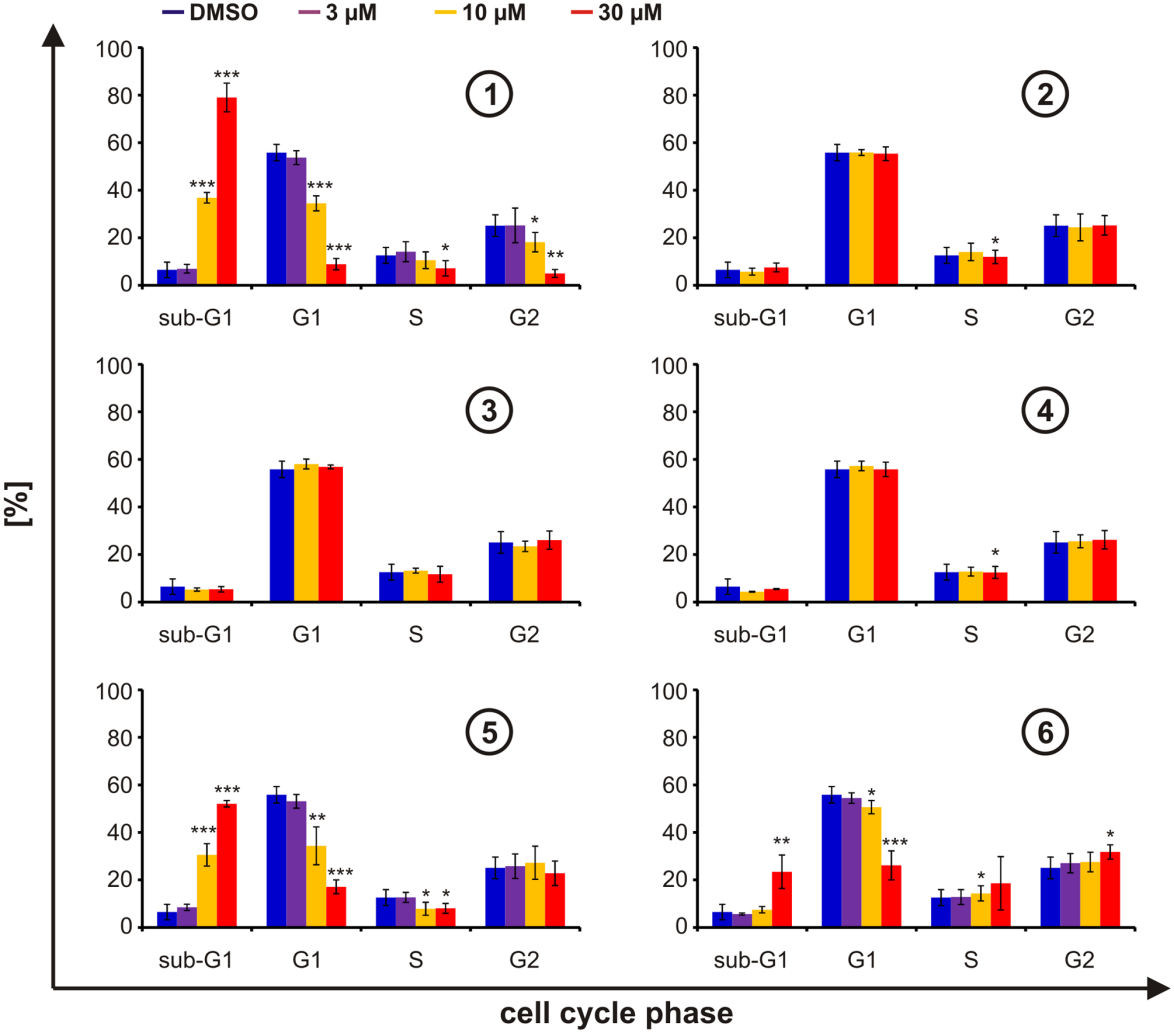
Cell cycle. HEK293 cells stably expressing Pdcd4_(39–91)_luc were incubated for 24 h with DMSO (blue) or increasing concentrations of compounds **1**, **2**, **3**, **4**, **5**, and **6** (3 μM (violet), 10 μM (yellow), and 30 μM (red)). Cell cycle analysis was performed after permeabilization using 7-AAD. Data are presented as means ± SEM of at least 3 independent experiments (* p<0.05, ** p<0.01, *** p<0.01).

As it was previously shown that stabilization of Pdcd4 can affect the cell cycle [14,25], we next evaluated if the compounds induce changes in cell cycle progression. To this end, we incubated cells for 24 h with the compounds, and then determined cell cycle phases *via* incorporation of 7-AAD in permeabilized cells. In line with our above-shown findings, compounds **2**, **3**, and **4** did not alter the cell cycle (Fig 5). Furthermore, compounds **1**, **5**, and **6** lead to a significant increase of cells in sub-G1 phase, which is indicative for enhanced cell death rather than for changes in cell cycle progression. Again, compound 1 showed the most pronounced effects, whereas compound 6 appeared least potent, compound 5 leading to intermediate changes.

**Fig 6:**
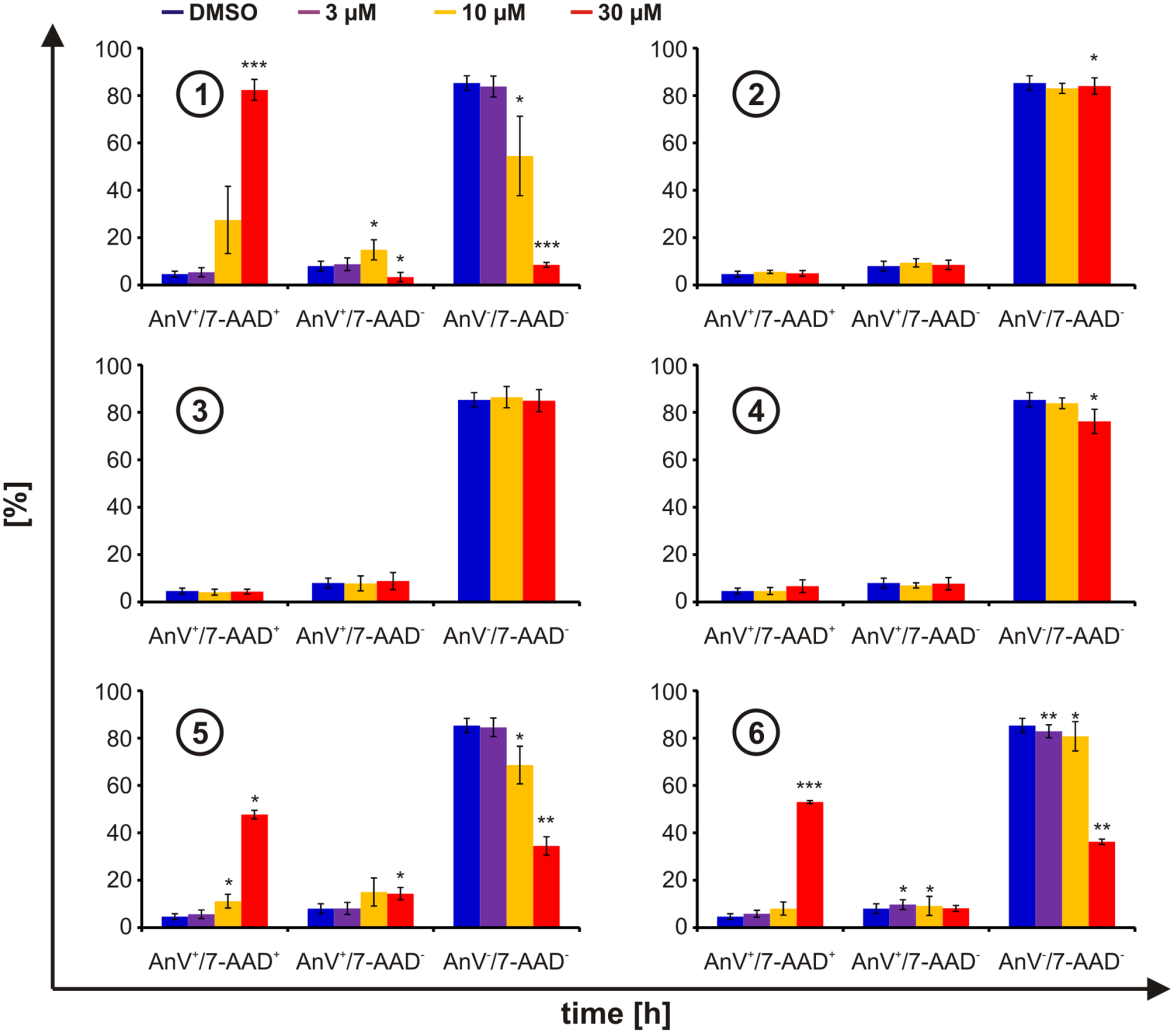
Viability. HEK293 cells stably expressing Pdcd4_(39–91)_luc were incubated for 24 h with DMSO (blue) or increasing concentrations of compounds **1**, **2**, **3**, **4**, **5**, and **6** (3 μM (violet), 10 μM (yellow), and 30 μM (red)). Cell death analysis was performed using Annexin V and 7-AAD co-staining. Data are presented as means ± SEM of at least 3 independent experiments (* p<0.05, ** p<0.01, *** p<0.01).

Since these observations pointed to toxicity rather than to proliferation inhibition, we next analyzed if cells underwent apoptotic or necrotic cell death in response to the compounds. Therefore, cells treated for 24 h with the compounds were stained with Annexin V and 7-AAD without permeabilization. Apoptotic cells should display phosphatidylserine on their membranes (AnV ^+^), yet still retain intact membranes (7-AAD ^-^), while necrotic cells should be both AnV ^+^ and 7-AAD ^+^ due to disruption of the cellular membranes. Again only compounds **1**, **5**, and **6** caused a marked decrease in viable cells (AnV ^-^/7-AAD ^-^) and a strong and concentration-dependent increase in necrotic cells (AnV ^+^/7-AAD ^+^) (Fig 6). The observed loss of viability nicely correlates with both the increase in the sub-G1 fraction as well as with the inhibition of proliferation. Yet, proliferation appears to be a more sensitive marker, likely due to the longer time frame of exposure. The loss of viability (Fig 6) coincides closest with the nonspecific luciferase reporter (Fig 3), which reflects the same incubation conditions. Interestingly, Pdcd4 stabilizing activities of compound **1**, **5**, and **6** (Fig 2) occurred at or slightly below the viability reducing concentrations (Fig 6). Thus, diaryl disulfides appear to stabilize Pdcd4 eventually inducing cell death.

### Computational structure activity relationship analysis

**Fig 7:**
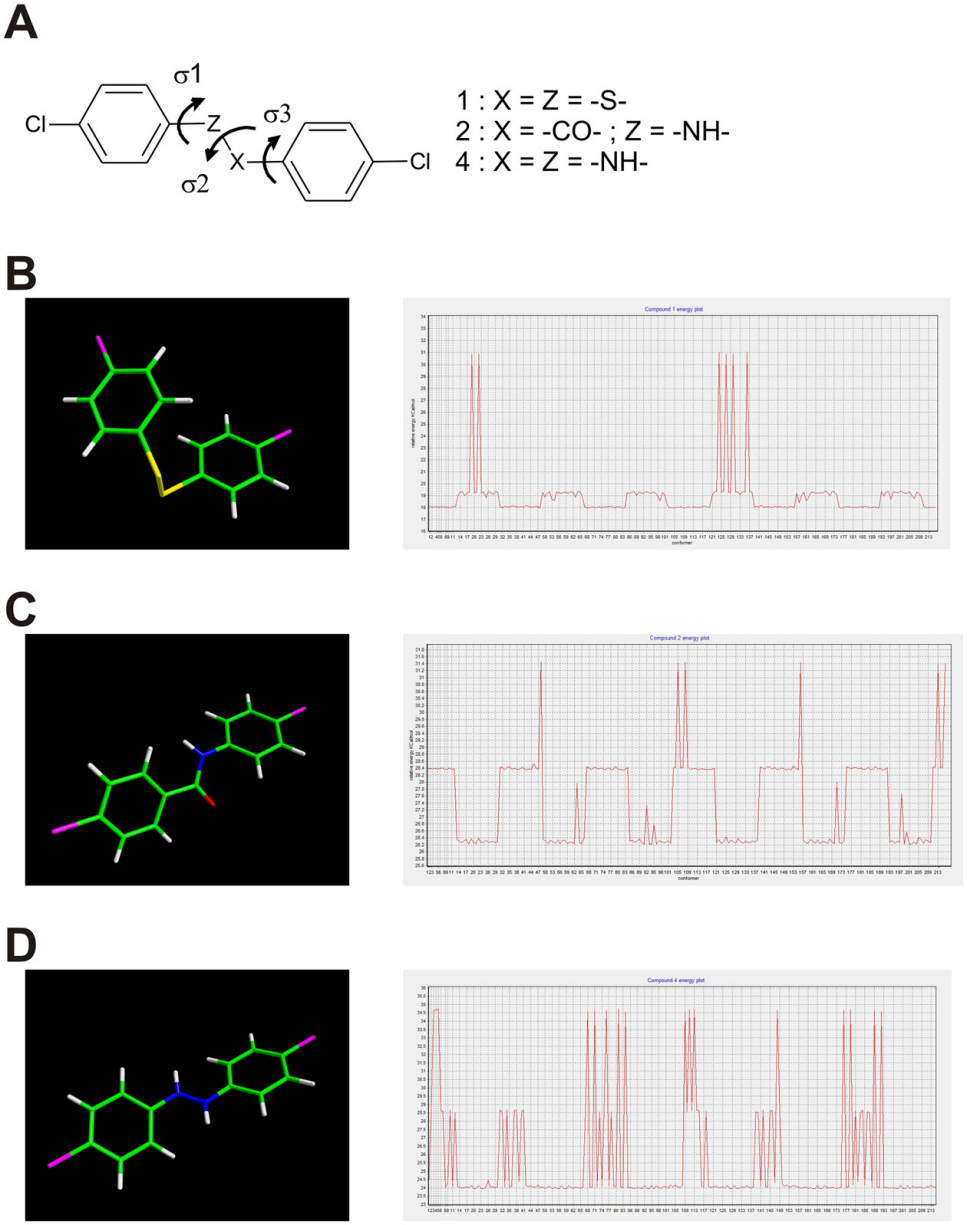
Conformational analysis of compounds 1, 2 and 4. (A) A systematic approach was chosen to explore the conformational space around the three rotable bonds. (B-D) Global minimum energy conformations (*left panel*) and energy plots (*right panel*) of compounds **1** (B), **2** (C), and **4** (D).

In an initial attempt to assess the influence of the conformational features of the linker region on the biological activity, we carried out a computational study on compounds **1**, **2**, and **4** by exploring the conformational space around the three rotatable bonds (σ1, σ2, σ3; Fig 7A).

The conformations generated and minimized for compound **1** can, for the most part, be placed into two very close clusters energy, thus indicating a high conformational flexibility. The global minimum energy conformation presents a folded structure (Fig 7B). In contrast, the energy plot shows that the conformations generated and minimized for compound **2** can be roughly grouped into two energy clusters with a higher energy difference compared to compound **1** indicating a lower conformational freedom. In this case, the global minimum was found as an extended conformation with the aromatic ring almost perpendicular. The conformations generated and minimized for compound **4** (Fig 7C), from the point of view of the energy are quite close to that of the global minimum but with a greater number of conformations at a higher energy content than what is observed for compound **1**. The global minimum was found as extended conformation with the two aromatic rings slightly inclined relatively to each other. Taken together, our computational study revealed a higher flexibility of compound **1**, due to the disulfide group, compared to the hydrazine (compound **4**), and even more so to the amide (compound **2**). Furthermore, compound **1** appears to possess a folded global minimum conformation, whereas compounds **2** and **4** appear almost extended, which may contribute to the difference in activity found in the compounds tested.

## Conclusions

In the present study we identified a novel stabilizer of tumor suppressor Pdcd4, 1,2-bis(4-chlorophenyl)disulfide (compound **1**), which not only stabilized Pdcd4 but also inhibited cellular proliferation. Interestingly, substituting the disulfide linker region completely abolished the Pdcd4 stabilizing and proliferation inhibitory activities, whereas modulation of the phenyl substituents only had a minor impact on the biological activity. Yet, it can be envisioned that other modifications could enhance the Pdcd4 stabilizing potential. The need of the disulfide linker, which was also supported by our computational study, implies a crucial role of this specific moiety for exerting the Pdcd4 stabilizing function of this compound. Nevertheless, further studies are needed to shed light on the molecular target of the diaryl disulfides mediating the Pdcd4 stabilizing effect, also exploring the exact chemical mode of interaction. Thus, while the compounds so far display rather low activities, it remains to be seen, if diaryl sulfide-based compounds can be optimized to yield more potent Pdcd4 stabilizers.
